# The Korean‐Lung Information Needs Questionnaire: Translation, validation and clinical implications in comprehensive pulmonary rehabilitation

**DOI:** 10.1111/crj.13487

**Published:** 2022-04-26

**Authors:** Sang Hun Kim, Ho Eun Park, Jin A Yoon, Yong Beom Shin, Myung‐Jun Shin, In Joo Kong, Ki Uk Kim

**Affiliations:** ^1^ Department of Rehabilitation Medicine, Biomedical Research Institute Pusan National University Hospital Busan South Korea; ^2^ Department of Rehabilitation Medicine, Biomedical Research Institute Pusan National University Hospital and Pusan National University School of Medicine Busan South Korea; ^3^ Department of Internal Medicine Pusan National University School of Medicine Busan South Korea

**Keywords:** chronic obstructive pulmonary disease, questionnaire, reliability, translation, validity

## Abstract

**Purpose:**

Chronic obstructive pulmonary disease (COPD) is characterized by airflow limitation and persistent respiratory symptoms. Several symptom‐related questionnaires have been validated to improve understanding for patient with COPD. We aimed to systematically translate the English version of the Lung Information Needs Questionnaire (LINQ) into Korean and to verify the reliability, validity and clinical implications in comprehensive pulmonary rehabilitation (PR).

**Methods:**

The original version of LINQ was translated into Korean by two translators and reviewed by the translation committee. It was then reverse translated back into English. The committee compared the reconciled version in Korean and the original version in English. A cognitive debriefing was performed on the pre‐final version, and a final version, K‐LINQ, was obtained. A test‐retest method for the analysis of reliability was performed within 2 weeks. Concurrent validity analysis was performed using Pearson correlation test of the K‐LINQ and other evaluation tools.

**Results:**

A total of 110 patients were enrolled. The length, readability, understanding and suitability of the questionnaire rated well in scores for face validity of 52 Korean patients with COPD. No significant correlation was derived from the total K‐LINQ and each domain with other scales such as mMRC, K‐CAT and K‐LCADL. The intra‐class correlation coefficient of total score K‐LINQ showed high reliability. Patients who attended PR showed significantly poor pulmonary function or more severe symptoms. In addition, a significantly lower score in total score and exercise domain of K‐LINQ were confirmed in the group of PR attendees.

**Conclusions:**

We translated the LINQ into Korean, implemented cross‐cultural adaptation and verified its validity and reliability. K‐LINQ can now be useful in various clinical and research fields in the Republic of Korea and could serve a complementary role and become an axis of successful treatment strategies, including a comprehensive PR programme.

## Graphical abstract

We translated the English version of the Lung Information Needs Questionnaire (LINQ) into Korean, implemented cross‐cultural adaptation and verified its validity and reliability. The length, readability, understanding and suitability of the questionnaire rated well in scores for face validity. The intra‐class correlation coefficient of total score K‐LINQ showed high reliability. Patients who attended pulmonary rehabilitation (PR) showed a significantly lower score in total score, and exercise domain of K‐LINQ was confirmed in the group of PR attendees.

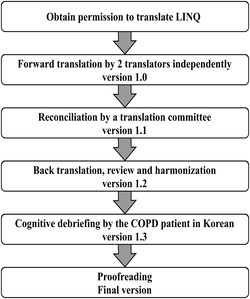

## 1 INTRODUCTION

Chronic obstructive pulmonary disease (COPD) is caused by increased airway resistance and damage to the lung parenchyma due to exposure to harmful particles or gases and is characterized by airflow limitation and persistent respiratory symptoms.^1^ Pulmonary rehabilitation (PR) is a comprehensive intervention based on thorough patient assessment and includes exercise training, education and behaviour changes. It aims to improve the physical and psychological condition of patients with chronic lung disease and is defined as a patient‐specific treatment that promotes long‐term implementation of health‐promoting behaviour.^2^ Regardless of the level of pulmonary function in COPD, worsening respiratory distress symptoms decrease physical function, limit activities of daily living and even accompany psychological problems, including depression and anxiety.^3^, ^4^ Therefore, patients with low health and psychological status should consider PR when symptoms worsen or limit their activities even with appropriate medical treatments.

A PR programme must include exercise and comprehensive interventions, including disease‐specific knowledge and self‐management.^5^ Therefore, there is a need to establish treatment plans through a verified evaluation of the need for education and information for patients that may be overlooked. Several symptom‐related questionnaires for patients with COPD have been validated.^6^, ^7^, ^8^ Among them, the Lung Information Needs Questionnaire (LINQ) is a unique self‐completion questionnaire for patients with chronic lung disease, which makes it easy for healthcare professionals to determine what information the patient needs.^9^ Therefore, quality of patient education can be measured through the LINQ as the outcome measure of comprehensive PR.^10^ The LINQ has already been translated and validated in Spanish and Italian, but not Korean.^11^, ^12^ This study aimed to perform the translation and cross‐cultural adaptation of the LINQ into Korean and to verify the reliability, validity and clinical implications thereof.

## 2 MATERIALS AND METHODS

This study comprised three parts: (1) translation and cultural adaptation of the original English version of LINQ into Korean; (2) test of reliability and validity of the K‐LINQ and (3) analysis of clinical implications in PR for patients with COPD.

### 2.1 Translation and cultural adaptation

**FIGURE 1:**
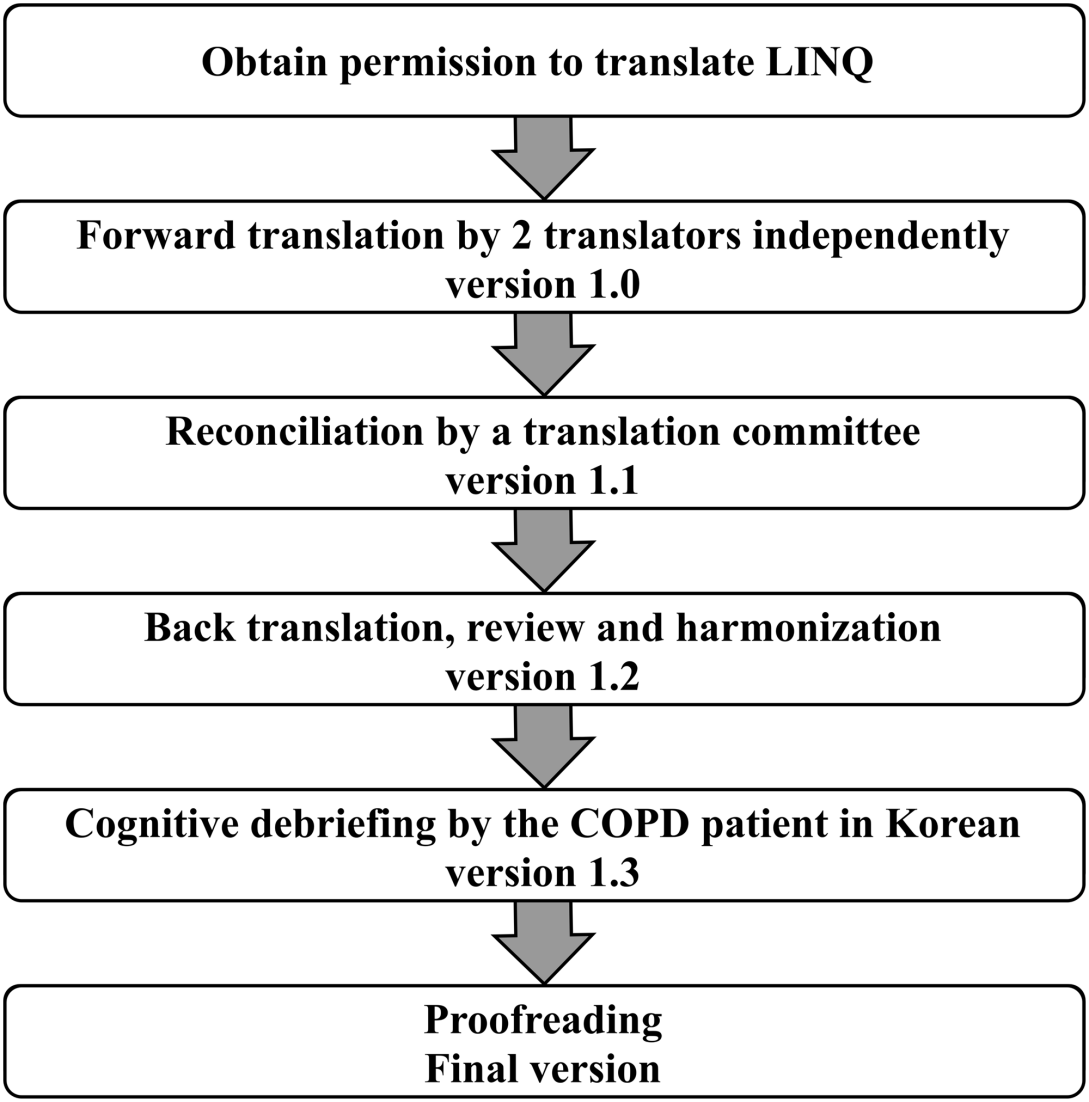
Flow chart of translation and cultural adaptation of the Lung Information Needs Questionnaire

Permission to translate the LINQ to Korean was obtained from the author. The translation and cultural adaptation were performed based on the International Society for Pharmacoeconomics and Outcomes Research (ISPOR) Patient‐Reported Outcomes Translation and Linguistic Validation Task Force guidelines (Figure 1).^13^

The original version of the LINQ was initially forward‐translated from English into Korean by two bilingual translators, independently, who were part of the medical team that participated in this study. The translation committee, comprising four experts including physicians, reviewed two translations and created the synthesized Korean version to resolve a few discrepancies. Another bilingual translator, who was non‐medical personnel, reverse‐translated the K‐LINQ into English. The translation committee compared the reconciled version in Korean and the original version, making the translations consistent. A cognitive debriefing was performed on the pre‐final version of the K‐LINQ for several Korean patients with COPD at Pusan National University Hospital. The final version of the K‐LINQ was subsequently established, after the ambiguous or confusing expressions were modified by the translation committee (Appendix S1).

### 2.2 Participants

A total of 110 patients with COPD were recruited between October 2020 and May 2021. The inclusion criteria were as follows: (1) diagnosis of COPD according to the Global Initiative for Chronic Obstructive Lung Disease (GOLD) guidelines^1^ and (2) age >40 years. Participants who had difficulty in completing the questionnaire in Korean were excluded.

### 2.3 Assessments

All participants completed the K‐LINQ, which consists of six domains: disease knowledge (four items); medicines (three items); self‐management (two items); smoking (three items); exercise (three items) and diet (one item). There are two types of questions: yes or no response type and multiple‐choice type. Scores range from 0 to 25, and the higher the score, the more information required. Several symptom‐related questionnaires, such as the Korean version of COPD Assessment Test (K‐CAT)^14^; the modified Medical Research Council (mMRC) dyspnea scale; the Korean version of strength, assistance with walking, rising from a chair, climbing stairs and falls questionnaire (K‐SARC‐F)^15^; and the Korean version of the London Chest Activity of Daily Living (K‐LCADL)^16^ scale were also conducted at their first visit.

Participants were asked to re‐respond to the K‐LINQ within 2 weeks, and a retest survey of 42 participants was collected. Information about age, sex, height, weight, body mass index, smoking history, the refined ABCD assessment tool,^17^ post‐bronchodilator forced vital capacity (FVC) and forced expiratory volume in 1 s (FEV_1_) and the number of outpatient visits to the department of pulmonary medicine or PR were collected from the medical chart review.

### 2.4 Validity and reliability

Face validity and concurrent validity were verified for the final version of K‐LINQ. Face validity is the determination of how adequately the survey questions or observations contained in the measurement tool contain the attributes or concepts that they intend to measure. This study recruited an individual sample of 52 participants who met the inclusion criteria, to evaluate the degree of clarity of understanding objectively and constructively and to determine whether there was any ambiguity or multiple ways to interpret the questions. Patients with COPD were surveyed on an eight‐item visual analogue scale (VAS) scale regarding the length, composition, readability, understanding and suitability of the questionnaire items. This was evaluated using a 100 mm VAS, and we defined 0 mm as *not usable at all* and 100 mm as *very usable*. The concurrent validity determined how much the value measured by K‐LINQ was consistent with the other questionnaires that were already validated.

A prospective evaluation was performed to verify the intra‐class correlation coefficient (ICC) for the test‐retest reliability. According to a test‐retest method for the analysis of reliability, 42 patients conducted the K‐LINQ and repeated the questionnaire within 2 weeks.

### 2.5 Statistical analysis

The baseline characteristics of patients are presented as absolute variables for categorical variables and means with standard deviations for numerical variables. For concurrent validity, the Pearson correlation test of K‐LINQ and other symptom‐relate questionnaires for COPD patients were analysed. The test‐retest reliability was measured using ICC for agreement. The group was divided according to the PR clinic visited, and independent *t* tests were performed on continuous variables which had normal distribution. A value of *p* < 0.05 was considered statistically significant. All data analyses were performed with IBM SPSS statistics software, version 22.0 for Windows (Armonk, NY, USA).

## 3 RESULTS

### 3.1 Baseline characteristics

A total of 110 patients with COPD participated in this study. The average age was 68.48 ± 6.94 years, and 91.8% were male. When the refined ABCD assessment tool was classified, group A accounted for the largest proportion, with 70 patients. The average number of outpatient visits to the department of pulmonary medicine and PR clinic was 22.01 ± 20.63 and 4.48 ± 5.42, respectively. Demographics and other assessments of patients are presented in Table 1. According to the refined ABCD assessment tool, total scores of the K‐LINQ were 7.81 ± 4.09 in the group A and 8.02 ± 3.26 in the group B. There was also a marginal difference in each domain score (Table 2).

**TABLE 1:** Characteristics of the subjects (*n* = 110)

	Value
Age (years)	68.48 ± 6.94
Sex (male, %)	91.8
Body Mass Index (kg/m^2^)	23.86 ± 3.05
Smoking history (pack year)	42.09 ± 19.43
ABCD assessment tool	
A	70 (63.6%)
B	39 (35.5%)
C	1 (0.9%)
D	0
mMRC dyspnea scale	1.42 ± 2.07
Hand grip strength (kg)	29.81 ± 16.51
K‐CAT score	5.99 ± 5.62
K‐SARC‐F score	0.27 ± 0.64
K‐LCADL score	15.98 ± 2.25
Pre‐bronchodilator FVC (L)	3.20 ± 0.69
Pre‐bronchodilator FVC (% of predicted)	75.23 ± 12.11
Pre‐bronchodilator FEV_1_ (L)	1.92 ± 0.52
Pre‐bronchodilator FEV_1_ (% of predicted)	63.39 ± 14.69
Pre‐bronchodilator FEV_1_/FVC ratio (%)	59.95 ± 9.92
Post‐bronchodilator FVC (L)	3.28 ± 0.69
Post‐bronchodilator FVC (% of predicted)	76.14 ± 12.19
Post‐bronchodilator FEV_1_ (L)	2.01 ± 0.65
Post‐bronchodilator FEV_1_ (% of predicted)	65.48 ± 14.44
Post‐bronchodilator FEV_1_/FVC ratio (%)	60.90 ± 9.92
Number of outpatient visits to the department of pulmonary medicine	22.01 ± 20.63
Number of outpatient visits to the department of PR (*n* = 27)	4.48 ± 5.42

*Note*: Values are reported as mean ± standard deviation or percent.

Abbreviations: mMRC, modified Medical Research Council; K‐CAT, Korean version of chronic obstructive pulmonary disease assessment test; K‐SARC‐F, Korean version of strength, assistance with walking, rising from a chair, climbing stairs, and falls questionnaire; K‐LCADL, Korean version of the London Chest Activity of Daily living scale; FVC, forced vital capacity; FEV_1_, forced expiratory volume in one second; PR, pulmonary rehabilitation.

**TABLE 2:** Average score of K‐LINQ and each domain according to the refined ABCD assessment tool

	Group A (*n* = 70)	Group B (*n* = 39)	Group C (*n* = 1)	*p* value
K‐LINQ total	7.81 ± 4.09	8.02 ± 3.26	7	0.775
Disease knowledge	1.22 ± 1.03	1.22 ± 0.91	2	0.996
Medicines	0.56 ± 0.82	0.46 ± 0.74	0	0.543
Self‐management	2.43 ± 1.86	2.63 ± 1.68	3	0.560
Smoking	0.18 ± 0.52	0.24 ± 0.58	0	0.531
Exercise	1.96 ± 1.18	1.96 ± 1.36	1	0.985
Diet	1.47 ± 0.74	1.51 ± 0.75	1	0.778

*Note*: Values are reported as mean ± standard deviation. The *p* value was analysed using the independent *T* test between group A and B. A participant for group D was not recruited in this study.

Abbreviation: K‐LINQ, Korean‐Lung Information Needs Questionnaire.

### 3.2 Validity of K‐LINQ

Face validity was performed to confirm the level of comprehension and cognitive equivalence of the translation for the newly translated K‐LINQ. A total of 52 Korean patients with COPD were included, and the average response of all questions exceeded 85 mm in the VAS (Table 3). The Pearson correlation coefficient was analysed for the concurrent validity between K‐LINQ and other symptom‐related questionnaires for COPD patients such as K‐CAT, mMRC, K‐LCADL and K‐SARC‐F. No significant correlation was derived from the total K‐LINQ and each domain with other scales. However, a significant positive correlation with age was confirmed in the total K‐LINQ (*r* = 0.218) and domain of self‐management (*r* = 0.259).

**TABLE 3:** Subject opinions regarding usability of the Lung Information Needs Questionnaire (*n* = 52)

		Value
1	Is the questionnaire suitable for assessing ‘understanding of COPD disease’?	85.92 (79.50–92.34)
2	Do you think the questionnaire asks about your understanding of COPD disease?	83.85 (77.46–90.23)
3	Do you think the length of the questionnaire is appropriate?	93.42 (96.05–100)
4	Are the questions on the questionnaire clearly stated?	91.77 (86.44–97.10)
5	Do you think the questionnaire is well organized?	94.27 (90.52–98.02)
6	Did you find it difficult to read and understand the questionnaire?	95.10 (91.30–98.89)
7	Did you find it difficult to fill out the questionnaire?	97.56 (95.32–99.79)
8	Is the design of the questionnaire appropriate?	96.08 (93.01–99.14)

*Note*: Values are reported as mean (95 % confidence interval) and rated on visual analogue scale (0 [*not usable at all*] to 100 [*very usable*]).

Abbreviation: COPD, chronic obstructive pulmonary disease.

### 3.3 Intra‐rater reliability of the K‐LINQ

To confirm intra‐rater reliability, we performed a retest within 2 weeks with some participants. Patients who consented to a visit within 2 weeks, and who had no COPD‐related outpatient visit within 2 weeks, were included. The ICC for 42 subjects exceeded 0.6, except for the diet domain, and *p <* 0.01 for all variables (Table 4).

**TABLE 4:** Test‐retest reliability of the Korean version of the Lung Information Needs Questionnaire (*n* = 42)

Domain	1st assessment	2nd assessment	ICC	*p* value
K‐LINQ total	7.98 ± 3.49	8.00 ± 3.47	0.742	0.000
Disease knowledge	1.24 ± 0.96	1.19 ± 0.83	0.826	0.000
Medicines	0.50 ± 0.63	0.60 ± 0.59	0.658	0.000
Self‐management	2.52 ± 1.49	2.71 ± 1.66	0.761	0.000
Smoking	0.19 ± 0.55	0.17 ± 0.49	0.877	0.000
Exercise	1.95 ± 1.27	2.17 ± 1.15	0.610	0.002
Diet	1.50 ± 0.77	1.17 ± 0.82	0.506	0.008

*Note*: Values are reported as mean ± standard deviation.

Abbreviations: ICC, intra‐class correlation coefficient; K‐LINQ, Korean‐Lung Information Needs Questionnaire.

### 3.4 Clinical implications in PR

A subgroup analysis was performed by dividing the subjects according to whether they visited the PR outpatient clinic. Total score of the K‐LINQ and item‐by‐item analysis was performed through an independent *t* test between the two groups. Regarding patient demographics, FEV_1_, mMRC, K‐CAT, K‐LCADL and K‐SARC‐F, it was confirmed that the group of PR attendees showed significantly lower function or symptom status. In addition, a significantly lower score was confirmed in the total K‐LINQ (*p* = 0.033) and the domain of exercise (*p* = 0.036) (Table 5).

**TABLE 5:** Comparison with PR attendance

	PR non‐attendee (*n* = 83)	PR attendee (*n* = 27)	*p* value
Age (years)	68.54 ± 7.00	69.03 ± 5.96	0.742
Body mass index (kg/m^2^)	24.02 ± 3.05	23.42 ± 3.29	0.411
Smoking history (pack year)	32.99 ± 22.52	44.44 ± 24.55	0.027*
mMRC dyspnea scale	1.16 ± 2.05	2.04 ± 1.60	0.046*
Hand grip strength (kg)	29.22 ± 17.07	32.03 ± 11.80	0.429
K‐CAT score	4.80 ± 4.00	9.38 ± 7.51	0.000**
K‐SARC‐F score	0.13 ± 0.38	0.40 ± 1.01	0.039*
K‐LCADL score	15.69 ± 1.51	16.70 ± 3.31	0.030*
Post‐bronchodilator FVC (% of predicted)	77.13 ± 12.91	75.81 ± 10.48	0.635
Post‐bronchodilator FEV_1_ (% of predicted)	67.47 ± 14.39	60.37 ± 14.68	0.030*
K‐LINQ total	8.34 ± 3.65	6.56 ± 3.90	0.033*
Disease knowledge	1.30 ± 0.98	1.00 ± 0.96	0.168
Medicines	0.53 ± 0.80	0.48 ± 0.75	0.782
Self‐management	2.66 ± 1.73	2.11 ± 1.87	0.161
Smoking	0.23 ± 0.59	0.11 ± 0.32	0.192
Exercise	2.07 ± 1.29	1.56 ± 1.01	0.036*
Diet	1.54 ± 0.69	1.30 ± 0.87	0.188

Abbreviations: PR, pulmonary rehabilitation; mMRC, modified Medical Research Council; K‐CAT, Korean version of chronic obstructive pulmonary disease assessment test; K‐SARC‐F, Korean version of strength, assistance with walking, rising from a chair, climbing stairs, and falls questionnaire; K‐LCADL, Korean version of the London Chest Activity of Daily living scale; FVC, forced vital capacity; FEV_1_, forced expiratory volume in one second; K‐LINQ, Korean‐Lung Information Needs Questionnaire.

* *p* < 0.05.

** *p* < 0.01.

## 4 DISCUSSION

We translated the LINQ into Korean, implemented cross‐cultural adaptation and verified its validity and reliability. K‐LINQ may, therefore, be useful in various clinical and research fields in the Republic of Korea. We also confirmed that each self‐report questionnaire could serve a complementary role and become an axis of successful treatment strategies.

In this study, translation and cultural adaptation of LINQ into Korean were performed in accordance with international standard guidelines.^17^ The structure of K‐LINQ is the same as the original version, which consists of 6 domains and 16 items, and the questionnaire requires a validity and reliability test after translation. However, since there were no Korean questionnaires investigating the degree of education or information, we compared them with symptom‐related questionnaires such as mMRC, CAT, SARC‐F and LCADL, which are commonly evaluated in COPD patients. We also analysed correlations with age, FEV_1_ and FVC. No significant correlation was identified with symptom‐related surveys. However, since K‐LINQ is a questionnaire with items of various categories in addition to symptoms, the lack of significant correlation is an expected result. This does not imply that the questionnaire itself is not valid. Although somewhat low correlation coefficients, total K‐LINQ (*r* = 0.218) and the domain of self‐management (*r* = 0.259) showed significant positive correlation with age. This may be because older people may have less access and understanding of disease information. No significant correlation was identified with FEV_1_, which suggests that the degree of symptoms is not the only factor affecting education or information acquisition.

Another important concern is the incomplete evaluation based on heterogeneous perception among users.^13^ Face validity was implemented to solve this and is related with judgements about the appearance and understanding of each item in the questionnaire. As K‐LINQ is a self‐completion questionnaire of patients, we investigated 52 COPD patients with face validity through cognitive debriefing. Our results show that most of the respondents answered that the questionnaire was appropriate, well understood and well structured. Therefore, we did not need to make any additional modifications to the K‐LINQ that was already verified.

In this study, test‐retest reliability was performed within 2 weeks to confirm intra‐rater reliability. Scores of disease knowledge, smoking, self‐management domains and total score of K‐LINQ show high internal consistency coefficients of 0.70 or more. A moderate ICC of 0.5–0.7 was identified in the medicine, exercise and diet domains.^18^ A rather low ICC in some domains is believed to be due to the outpatient department process. Although the period was only 2 weeks, this may have resulted from the fact that there were some patients who first received inhaler prescriptions and training in outpatient clinics, and some patients who obtained nutritional and exercise‐related information at the PR clinic. In other words, the researchers could not completely control the timing patient evaluations, so differences would inevitably occur before and after visiting the outpatient clinic.

We found that K‐LINQ was a valid and reliable questionnaire and a self‐questionnaire that would be useful in patients diagnosed with COPD. Since December 2016, PR for chronic lung disease has become eligible for reimbursement, and attention and demand for PR have increased in the Republic of Korea. Validated assessments in terms of self‐education of COPD patients are necessary for comprehensive PR. CAT, mMRC, LCADL and Saint George's Respiratory Questionnaire (SGRQ), which have been previously validated and used for COPD patient evaluation in the Republic of Korea, are mainly based on symptoms, making it difficult to determine the patient's level of understanding of the disease. Meanwhile, LINQ is a questionnaire developed to measure the need for disease‐related information in COPD patients. It is easy for patients to understand and takes only 6 min to complete in the waiting room. As it has been translated into 18 languages and validated in several languages, it is useful in clinical practice. In addition, it is being used in various research fields, such as to determine the educational effect of a comprehensive elderly rehabilitation programme, the importance and delivery mode of education in PR,^10^, ^18^, ^19^, ^20^, ^21^ the gender differences in information needs^22^ and the effect of pharmacist‐led education.^23^

PR is suggested as an essential non‐pharmacological treatment for successful disease management in chronic lung diseases such as COPD.^24^ There are several barriers to successful PR, so the attendance rate remains low.^25^ Several multidisciplinary strategies are needed to lower re‐hospitalization and prevent acute exacerbation through comprehensive PR.^26^ Among them, patient education, self‐management and smoking cessation are essential factors, and it is helpful to establish an effective treatment strategy if the physician can quantitatively evaluate how much the patient knows. From this point of view, the differences between the two groups were compared according to whether they participated in PR. There were statistically significant differences between the PR non‐attendee and PR attendee groups in smoking history, post‐bronchodilator FEV_1_, mMRC, total score of K‐CAT, SARC‐F and K‐LCADL (Table 5). This means that patients with more severe symptoms and decreased function are referred to the PR clinic.

Similar to the results of previous study,^27^ in the K‐LINQ total score and exercise domain, lower scores were shown in the PR attendee group. It means that the patient was able to obtain additional information including physical activity and exercise from the physician by registering for the PR programme. On the other hand, there were also differences with other previous studies.^5^, ^24^ While previous studies showed that the information needs were significantly improved in all domains of LINQ other than smoking, this study showed no significant difference in the other domains except for exercise. This can be explained by the cultural background of South Korea that medical information access through the Internet is easy, and the national health insurance system is well established. Therefore, since the patients' information needs are not high even before PR, the reduction in information needs after PR is not statistically significant. The history of reimbursement of PR for patients with COPD in South Korea is not long, about 5 years. The significance of reducing the need for information in the exercise domain is a result of disproving the intervention in the field of rehabilitation medicine, which has not been enough until now. At the same time, this result suggests that the direction of PR for patients with COPD in South Korea in the future. Ultimately, it should be considered that a more comprehensive programme strategy through a multidisciplinary approach is needed in the future and that K‐LINQ can be used to establish this.

This study has several limitations. First, there was a small sample size, especially in group C, D of ABCD assessment tool. This may be because the higher the severity, the lower the willingness to participate in the questionnaire, the difficulty in visiting the hospital frequently due to severe dyspnea or that the patient is hospitalized. In addition, since this was a single centre study of patients visiting our tertiary referral hospital, there is a selection bias that cannot represent all COPD patients. Patients who often attend our tertiary hospital are likely to have systematically obtained a lot of COPD‐related information. In a future study, it will be necessary to determine the usefulness of K‐LINQ as an evaluation tool to confirm the effectiveness of comprehensive PR in all COPD patients.

## 5 CONCLUSIONS

**FIGURE 2:**
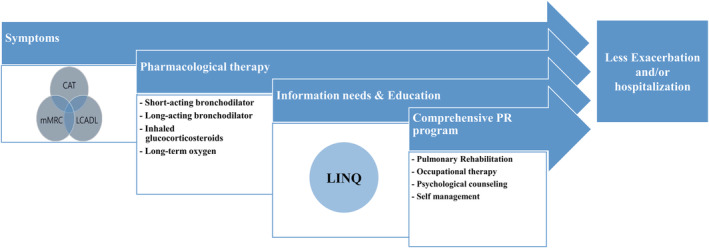
Components and goals of a successful treatment strategy in patients with COPD CAT, COPD Assessment Test; mMRC, modified Medical Research Council; LCADL, London Chest Activity of Daily Living; PR, pulmonary rehabilitation; LINQ, Lung Information Needs Questionnaire

We translated LINQ into Korean, implemented cross‐cultural adaptation and verified its validity and reliability. K‐LINQ can be useful in various clinical and research fields in the Republic of Korea, and it was confirmed that each self‐report questionnaire could serve a complementary role and become an axis of successful treatment strategies, including a comprehensive PR programme (Figure 2).

K‐LINQ is registered on the official website with the permission of the original author (http://www.linq.org.uk/LINQDownload.htm). There is no restriction on access to the homepage, and clinicians and researchers can freely download and use it in various areas related to COPD patients.

## CONFLICT OF INTEREST

The authors report no conflict of interest in this work.

## ETHICS STATEMENT

This study was approved by the Institutional Review Boards of Pusan National University Hospital (IRB No. 2010‐009‐096), and all participants provided written‐informed consent.

## AUTHOR CONTRIBUTIONS

SHK, HEP, JAY and KUK contributed to protocol development, data analysis and translation and drafted the manuscript. YBS and MJS contributed to study conception and participated in its coordination. IJK, SHK and HEP contributed to data collection. SHK and KUK designed the study and contributed to the overall management of the study. All authors reviewed the manuscript and approved the final version of the manuscript.

## Supporting information


**Appendix S1.** Survey on the need for information on chronic obstructive pulmonary disease in Korea

## Data Availability

The data that support the findings of this study are available in the supporting information.
