# Supplementary material for: The Korean‐Lung Information Needs Questionnaire: Translation, validation and clinical implications in comprehensive pulmonary rehabilitation
Source: Clin Respir J. 2022 Apr 26;16(5):343–51. doi: 10.1111/crj.13487 (PMC9366590; doi:10.1111/crj.13487)
Supplement: Supplementary file 1 — Appendix S1. Survey on the need for information on chronic obstructive pulmonary disease in Korea [file CRJ-16-343-s001.docx]

**Appendix 1. Survey on the need for information on chronic obstructive pulmonary disease in Korea**

1. Do you know the name of your lung disease ?

Yes     □

no     □

2. Have you heard from your medical staff ( doctors , nurses ) and therapists about how this disease can affect your lungs ?

Yes     □

no     □

3. Have you been informed by your medical staff ( doctors , nurses ) and therapists about what might happen in the future ?

Yes     □

no     □

4. Choose a situation from the list below that you think could happen to you within a few years . Please choose only one .

 My illness is being treated , and it will get better in the future .     □

 My illness is being treated, and it will remain similar in the future .   □

 My condition will only get worse .       □

 I do n't know .         □

5. Did the medical staff ( doctor , nurse ) explain why you should use an inhaler or medication ?

Yes     □

no     □

6. Do you try to use your inhaler or medication exactly as instructed by your medical staff ( doctors , nurses ) ?

Yes     □

no     □

7. Are you satisfied with the information you received from medical staff ( doctors , nurses ) regarding inhalers or medications ? Please choose only one .

 I understand everything you need to know .      □

 I understand what you have been told , but I want more information .    □

 I'm a little confused about the drug .       □

 I am very confused about the drug .       □

8. What is the most appropriate method for performing breathing problems ? ( eg , applying the inhaler more often ) Choose one .

 I received instructions from the medical staff ( doctors , nurses ) and received educational materials .   □

 I was given instructions , but I did n't receive any training materials .     □

 I haven't heard the instructions , but I know what to do .    □

 I did n't hear the instructions and I do n't know what to do .    □

9. Have you ever been told when to call an ambulance if you have trouble breathing ? Please choose only one .

 I received instructions from the medical staff ( doctors , nurses ) and received educational materials .   □

 I was given instructions , but I did n't receive any training materials .     □

 I haven't heard the instructions , but I know what to do .    □

 I did n't hear the instructions and I do n't know when to call an ambulance .  □

10. What is your condition ? Please choose only one .

 I have never smoked . (Go to question 13 )      □

 He smoked in the past, but is now a smoker . (Go to question 13 )   □

 Currently smoking . _ (Go to question 11 )       □

11. Did your medical staff ( doctors , nurses ) and therapists advise you to quit smoking ?

Yes     □

no     □

12. Did the medical staff ( doctors , nurses ) help you quit smoking ? ( Example : nicotine gum , patches , information on smoking cessation clinics , etc. )

Yes     □

no     □

13. Have you ever been advised by your medical staff ( doctors , nurses ) to be physically active ? ( Example : walking , brisk walking , other exercise ) _

Yes     □

no     □

14. Did your medical staff ( doctors , nurses ) and therapists tell you about the appropriate amount of physical activity ? ( Example : walking , brisk walking , other exercise ) _

 I told you and I know what to do .      □

 Let me know but I'm not sure what to do .          □

but I ca n't get it to work .              □

 did not inform                       □

15. How much physical activity do you do ?

 Do as little as possible .        □

 try to do _        □

Do as much as you can .               □

16. Did the medical staff ( doctors , nurses ) tell you about your diet or diet ? Check all that apply .

 Eat small meals in multiple portions _                       □

 ( Example : 6 small meals rather than 3 large meals ) _ _ _

 lose or gain weight               □

 eating healthy food               □

 did n't tell me        □

17. Do you have any questions or comments about your lung disease ? If yes , please fill out the space below .

18. Do you live alone ?

Yes     □

no     □

19. Gender :    □ Male   □ Female

20. What is your year of birth ?   19 _ _

**Appendix 1. 한국판 만성폐쇄성폐질환에 대한 정보 필요성 설문**

1. 당신의 폐 질환명을 알고 계십니까?

예 □

아니오 □

2. 의료진(의사, 간호사)과 치료사에게 이 질환이 당신의 폐에 어떤 영향을 미칠 수 있는지에 대한 설명을 들었습니까?

예 □

아니오 □

3. 의료진(의사, 간호사)과 치료사에게 장래에 일어날 수 있는 상황에 대한 설명을 들었습니까?

예 □

아니오 □

4. 아래의 항목 중 몇 년 이내에 당신에게 일어날 수 있다고 생각되는 상황을 고르십시오. 한 가지만 고르십시오.

내 병은 치료 중이고, 앞으로는 더 나아질 것이다. □

내 병은 치료 중이고, 앞으로도 비슷한 상태를 유지할 것이다. □

내 상태는 더 나빠질 것이다. □

잘 모르겠다. □

5. 의료진(의사, 간호사)이 당신이 흡입기나 약제를 사용해야 하는 이유를 설명해 주었습니까?

예 □

아니오 □

6. 당신은 의료진(의사, 간호사)에게 교육 받은 대로 정확하게 흡입기나 약제를 사용하려고 노력합니까?

예 □

아니오 □

7. 당신은 흡입기나 약제 관련하여 의료진(의사, 간호사)에게 받은 정보에 대해 만족하십니까? 한 가지만 고르십시오.

알아야 할 내용은 모두 이해하고 있다. □

들은 내용은 이해하고 있지만 더 많은 정보를 원한다. □

약제에 대해 조금 헷갈린다. □

약제에 대해 많이 헷갈린다. □

8. 호흡이 나빠질 때 시행할 방법에 가장 적절한 항목은 무엇입니까? (예: 흡입기를 더 자주 적용하기) 한 가지만 고르십시오.

의료진(의사, 간호사)에게 지시 사항을 들었고, 교육 자료도 받았다. □

지시 사항을 들었으나 교육 자료는 받지 못했다. □

지시 사항을 듣지 못했지만 어떻게 해야 하는지 알고 있다. □

지시 사항을 듣지 못했고 어떻게 해야 하는지 모르겠다. □

9. 호흡이 나빠질 경우 구급차를 언제 불러야 할지 들은 적이 있습니까? 한 가지만 고르십시오.

의료진(의사, 간호사)에게 지시 사항을 들었고, 교육 자료도 받았다. □

지시 사항을 들었으나 교육 자료는 받지 못했다. □

지시 사항을 듣지 못했지만 어떻게 해야 하는지 알고 있다. □

지시 사항을 듣지 못했고 언제 구급차를 불러야 하는지도 잘 모르겠다. □

10. 당신은 어떤 상태입니까? 한 가지만 고르십시오.

흡연해본 적이 없다. (13번 문항으로) □

과거에 흡연을 하였으나 현재는 금연한 상태다. (13번 문항으로) □

현재 흡연 중이다. (11번 문항으로) □

11. 의료진(의사, 간호사)과 치료사가 당신에게 금연을 권고하였습니까?

예 □

아니오 □

12. 의료진(의사, 간호사)이 금연을 위해 도움을 주었습니까? (예: 니코틴 껌, 패치 제공, 금연 클리닉 안내 등)

예 □

아니오 □

13. 의료진(의사, 간호사)에게 신체 활동을 하도록 권고 받은 적이 있습니까? (예: 걷기, 빨리 걷기, 그 외 운동)

예 □

아니오 □

14. 의료진(의사, 간호사)과 치료사가 적절한 신체 활동량에 대해 알려 주었습니까? (예: 걷기, 빨리 걷기, 그 외 운동)

알려주었고 무엇을 해야하는지 알고 있다. □

알려주었지만 무엇을 해야하는지 잘 모르겠다. □

알려주었지만 나는 그것을 해낼 수 없다. □

알려주지 않았다. □

15. 신체활동을 얼마나 하십니까?

가능한 적게 한다. □

하려고 노력은 한다. □

할 수 있는 만큼 최대한 한다. □

16. 의료진(의사, 간호사)이 식단이나 식사에 대해 이야기해주었습니까? 해당되는 항목에 모두 표시하십시오.

적은 양의 식사를 여러 번 나누어 먹기 □

(예: 많은 양의 식사 3회 보다는 적은 양의 식사 6회)

체중을 줄이거나 늘리기 □

건강한 음식을 섭취하기 □

이야기 해주지 않았다. □

17. 당신의 폐질환에 대한 질문이나 의견이 있습니까? 있다면, 아래의 칸에 작성해주십시오.

18. 혼자 거주 하십니까?

예 □

아니오 □

19. 성별: □ 남성 □ 여성

20. 출생연도가 언제입니까? 19 _ _
